# Understanding the multilevel factors influencing the implementation of digital health interventions for supportive care in Adolescents and Young Adult (AYA) cancer survivorship: determinants of adopting mindfulness-based mobile applications

**DOI:** 10.1186/s43058-024-00612-w

**Published:** 2024-07-17

**Authors:** Gary Kwok, Angela Senger, Archana Sharma, Ivelisse Mandato, Katie A. Devine

**Affiliations:** 1 Cancer Prevention Precision Control Institute, Center for Discovery & Innovation, at Hackensack Meridian Health, Nutley, NJ 07110 USA; 2 Department of Pediatrics, Rutgers Cancer Institute of New Jersey, New Brunswick, NJ 08901 USA

**Keywords:** Psycho-Oncology, Adolescents and Young Adults, Cancer Survivorship, Digital Health/mHealth

## Abstract

**Background:**

Adolescents and Young Adult (AYA) cancer survivors are at risk for psychological distress due to their unique developmental and medical needs. Healthcare providers can leverage the convenience and appeal of technology to provide supportive care for this vulnerable population. Using evidence-based mindfulness-based mobile interventions as a case example, the goal of this study was to identify key patient-, provider-, and organization-level barriers and facilitators to supportive care and implementing digital health interventions in AYA survivorship care.

**Methods:**

Twenty semi-structured interviews were conducted with stakeholders including AYA survivors (*n* = 10; between 18–29 years old) and clinical providers and administrators (*n* = 10). Interviews were transcribed and deductively mapped using the Consolidated Framework for Implementation Research (CFIR) and Theoretical Domains Framework (TDF) complementary frameworks.

**Results:**

Results indicated that factors like cost and patients’ needs and resources were prevalent among both survivors and providers. There were key differences between providers and AYA survivors. Providers’ adoption and promotion of digital health interventions were influenced most strongly by contextual factors, including available resources (Inner Setting), culture (Outer Setting), and networks and communications (Outer Setting). On the other hand, survivors emphasized individual and intervention-related factors; they reported that social influence and knowledge influenced their adoption and use of digital health interventions, including meditation apps.

**Conclusions:**

These results identified barriers and facilitators to the adoption of supportive care digital health interventions from multiple stakeholders. Results can be used to guide the development of implementation strategies to improve the uptake of digital health interventions in survivorship care, ultimately improving the psychosocial well-being of AYA cancer survivors.

**Supplementary Information:**

The online version contains supplementary material available at 10.1186/s43058-024-00612-w.

Contributions to the literature:

- Identification of multilevel implementation challenges and subsequent potential tailored implementation strategies could be used as a blueprint for other digital health implementations in other clinical settings.
- Demonstration of the use of complementary determinant frameworks (i.e., CFIR + TDF) shows how combining complementary frameworks provides the opportunity to address a wider range of factors that may align better with different stakeholders’ needs.

## Background

Adolescent and young adult (AYA) survivors of childhood cancer are recognized as a vulnerable group with unique emotional, social, and practical needs due to the intersection of cancer survivorship and normal developmental processes [1]. While the developmental transition can become overwhelming and stressful in and of itself, managing cancer survivorship care during this time can be extremely difficult [2]. Studies suggest that AYA survivors of childhood cancer are at increased risk of poor mental health compared to non-cancer peers [3]. While 20–25% of AYA survivors of childhood cancer report experiencing impaired mental health [4, 5], many more experience subclinical levels of distress potentially impacting their daily functioning. However, most AYA cancer survivors do not receive formal psychological treatment [6, 7], suggesting that alternative interventions are needed to reach and support more survivors.

Routine medical follow-up care offers an opportunity to assess for and refer or treat mental health problems among childhood cancer survivors [8]. Providers such as oncologists and nurses can leverage these opportunities to provide psychosocial and supportive care during follow-up visits. For example, a social worker or psychologist might assess survivors’ psychosocial functioning and provide a psychosocial care plan during their clinic visits [9]. Furthermore, providers can promote supportive care through digital health interventions as they are cost-effective [10] and efficacious in alleviating stress and improving well-being among young adult cancer survivors [11]. Most AYA cancer survivors own a mobile phone, regularly use mobile apps, and are interested in accessing supportive care information via mobile apps [12, 13] which make digital health interventions appealing options.

Complementary and Alternative Medicine (CAM) such as meditation and other mindfulness modalities have become increasingly popular [14] and have shown efficacy in improving psychological distress among cancer patients [15]. The Society of Integrative Oncology Clinical Practice Guidelines have recommended meditation as a practice for reducing anxiety and stress [16]. Mindfulness-based digital health interventions could offer alternative options for supportive care in AYA survivorship clinical settings without interfering with the clinic workflow. Unlike traditional mindfulness interventions, mobile apps can be tailored to the individual user, with brief practice (e.g., 10 minutes a day) and goals adjusted to fit one’s schedule [17]. Mobile apps have also been demonstrated to be an effective delivery medium for mindfulness training [17], and have demonstrated efficacy in improving mental health outcomes across the general and cancer populations [17–20]. Evidenced-based mindfulness mobile apps have been demonstrated to be a good alternative to address AYA cancer survivors’ unmet psychosocial needs [21]. Providers can support patients by leveraging the convenience (e.g., making simple recommendations/referrals) and appeal of technology (e.g., AYA as “digital natives” [22]).

However, it is difficult to translate and implement research-based interventions into actual clinical practice [23], especially digital health interventions in healthcare settings [24, 25]. Digital health interventions have to be embedded in the workflows and tools they regularly use for sustaining adoption [24]. When removing digital health interventions from controlled trials to real-world settings, low uptake and even abandonments become common [24, 25]. Thus, it is important to utilize systematic approaches to guide the process of implementing these interventions [26]. More specifically, there is a need for a systematic process to plan for implementation with the consideration of determinants, mechanisms, and strategies for effecting change [27]. One of which is to develop effective implementation strategies, that is, approaches or techniques that are used to enhance the adoption, implementation, sustainment, and scale-up (or spread) of the intervention [28].

To develop effective implementation strategies, we first need to understand the determinants (i.e., barriers, obstacles, enablers, and facilitators) within the local context [29, 30], which is often multilevel. Thus, the goal of this study is to identify potential barriers and facilitators to adopting digital health interventions for supportive care in AYA survivorship care (i.e., acceptability and adoption). Using mindfulness-based mobile app interventions as a case example, results can guide the development of implementation strategies to improve the uptake of digital health interventions for supportive care in practice, ultimately improving the psychosocial well-being of AYA cancer survivors [27]. Focusing on mindfulness-based mobile app interventions as a case example allows participants to concretely describe both digital delivery and the intervention content.

## Methods

### Theoretical approach

A multiple complementary framework approach was employed in this study. The Consolidated Framework for Implementation Research (CFIR) and Theoretical Domains Framework (TDF) are both well-operationalized, multi-level, theory-driven implementation determinant frameworks [31]. Birken and colleagues have extensively described the similarities between the CFIR and the TDF in terms of purpose, level, degree of theoretical heritage, and operationalizability [31]. For this study, the rationale behind using two complementary frameworks is, while CFIR [32] includes five main domains: intervention characteristics (e.g., adaptability), outer setting (e.g., patient needs and resources), inner setting (e.g., culture), process (e.g., planning), and characteristics of individuals (e.g., self-efficacy; focuses on individual-level constructs), TDF [33, 34] provides an additional focus on individual-level behavior change factors (i.e., Capability – *Knowledge*, Motivation – *Reinforcement*, Opportunity – *Social Influences*). Stakeholders, especially AYA cancer survivors, are expected to focus more on individual-level factors as they are more relevant to them. Lastly, both CFIR and TDF have been applied as determinant frameworks to a variety of studies exploring barriers and facilitators of intervention adoption including identifying determinants of successful implementation of evidence-based practices in public health agencies [35] and engaging pregnant women in smoking cessation [36]. Together, these frameworks provided a multilevel perspective to understand these important factors for implementation.

### Sampling and setting

A combination of convenience and snowball sampling strategy was used to select participants. Ten (*n* = 10) AYA cancer survivors were referred from a previous meditation mobile app study within the cancer survivorship clinic at the Rutgers Cancer Institute of New Jersey. The Rutgers Cancer Institute of New Jersey is the only NCI-designated comprehensive cancer center in New Jersey, and the survivorship clinic provides long-term evaluation, support, and health education for childhood cancer survivors. The clinic is staffed with a comprehensive team of specialists including oncologists, a nurse practitioner, a psychologist, and a social worker. We also sent recruitment materials to survivors who had participated in a recently completedly meditation mobile app study and agreed to be contacted for future research opportunities. We reached out to those survivors because we were interested in learning from survivors who have experience with meditation mobile apps. Inclusion/exclusion criteria include: 1) Current age 18–29 years (to capture emerging adults who are legally responsible for their own healthcare); 2) No documented or self-reported cognitive impairment that would prevent completion of the interview; 3) English speaking; and 4) Owns a mobile phone. For clinical providers and administrators, the research team generated a preliminary list of potential stakeholders at Rutgers. Participants would also give referrals/suggestions. A total of 10 potential participants were recruited for the study. Inclusion/exclusion criteria include: 1) Fulltime employee and 2) Currently working or have worked with AYA cancer survivors in a professional capacity. All providers and administrators were employees at Rutgers in various departments, including the Department of Pediatric Hematology/Oncology and Patient Experience. Any role was eligible (e.g., physician, nurse, psychologist, social worker), with the ideal participant having clinical experience that would lend insights into structural barriers to implementing digital health interventions/meditation mobile apps in patient care. In total, *N* = 20 participants were recruited for the study (no refusal or dropout).

All potential participants were sent an introductory email, outlining the purpose of the study and inviting their participation. Participants who expressed interest were scheduled for an interview with GK (Postdoc; Male) and IM (Research Assistant; Female), which was conducted via Zoom. Both interviewers had an interest in digital health and had prior experience and received training in conducting interviews. All interviews took place between April and September 2022. The interviews lasted between 17 and 49 minutes with an *M* = 29.15 minutes (*SD* = 9.10).

### Data collection and analysis

Perspectives of key informants were sought via semi-structured interviews. The semi-structured interview guide (see Supplementary material) was adapted from previous research by Hwang and colleagues designed to understand multilevel determinants of clinicians’ decision-making on adopting imaging for prostate cancer [37]. The interview questions were open-ended to elicit implementation strategies by identifying relevant factors within the CFIR + TDF frameworks (see Theoretical approach). In addition, Wienert and Zeeb’s [38] CFIR’s construct definitions were adapted to better reflect digital health app-focused implementation factors.

The interview guide was developed using an iterative process; first, the first author (GK) compiled a list of potential interview questions based on the literature review. The questions were then paired with the CFIR + TDF frameworks; questions were modified to reflect the definitions and the goal of each construct. The senior author (KAD) and medical expert (AS; AYA oncologist) on the team then reviewed and gave feedback on the questions. The process was repeated until an agreement was achieved. The structure of the interview guide began with a list of general questions regarding mental health and supportive care in AYA cancer survivorship to assess the context for the use of digital health interventions. The CFIR + TDF questions began at the individual level and progressed toward hospital- and structural-level constructs and ended with intervention-specific questions. The sequence of the questions mirrored the multilevel structure of the CFIR framework.

Interviews were audio recorded and transcribed verbatim; de-identified transcripts were then analyzed using NVivo qualitative data analysis software. A total of three team members conducted and analyzed data. First, the transcripts were independently read and coded using content analysis [39, 40]; content analysis is frequently used by health researchers to interpret interview data [41]. The coding process itself involved interpreting and assigning interview responses into the coding schema and they would be deductively mapped onto the CFIR + TDF frameworks. The coders would then meet to discuss and reconcile any discrepancies in the mapped codes. Field notes were also used to reconcile discrepancies. Additional inductive coding was done to identify other relevant factors outside of the current frameworks, which could potentially inform future implementation strategy development.

### Ethics and governance

This study received ethical approval from the Rutgers Biomedical and Health Sciences Institutional Review Board (IRB# Pro2021001403). Informed consent to participation was obtained from all participants. Anonymity was protected using generic descriptors throughout and redacted identifiable information when necessary.

## Results

Fifty percent of the AYA survivors and 90% of the providers/administrators identified as females. While all providers were non-Hispanic Whites, 70% of the patients were non-Hispanic Whites. A variety of clinical and administration positions were recruited including *n* = 3 oncologists, *n* = 2 registered nurses, *n* = 3 psychosocial providers, and *n* = 2 others (i.e., administrator in patient experience and child life specialist). While 80% of the providers have advanced degrees, all AYA participants had either graduated or were currently enrolled in a university.

The overarching question posed to providers addressed facilitators and barriers they might encounter related to promoting digital health interventions as supportive care measures in the clinical setting (i.e., survivorship care). Survivors were asked questions regarding what factors may influence their adoption and use of mobile apps for supportive care. Table 1 presents the frequency of factors/themes that emerged for providers and patients. *Patient Needs and Resources* (Outer Setting), *Cost* (Intervention Characteristics), *Evidence Strength and Quality* (Intervention Characteristics), and *Reinforcement* (Motivation) emerged as the factors most frequently endorsed by the two groups. Several notable differences emerged. While providers reported greater importance of *Available Resources* (Inner Setting), *Culture* (Inner Setting), and *Networks and Communications* (Inner Setting), survivors emphasized the importance of *Social Influences* (Opportunities).

**Table 1:** Frequency of factors mentioned by providers and patients (*N* = 20)

	Provider (*n* = 10)			Patient (*n* = 10)		
Domains/Constructs	n^a^	Barrier	Facilitator	n^a^	Barrier	Facilitator
Outer						
Patient Needs & Resources	10	–	Understanding patients' needs informs direction of providers' practice	10	–	Coping with late effects increases help seeking
Culture	8	Lack of established culture overlooks supportive care	Established culture drives orientation toward supportive care	–	–	–
Inner						
Available Resources	9	–	Existing resources and personnel make supportive care easy	–	–	–
Networks & Communications	7	–	Formal and informal communications help direct goals of patient care	–	–	–
Motivation		–				
Reinforcement	8	–	News and updates help providers stay informed	7	–	Positive interaction with intervention reinforces usage
Capability						
Knowledge	–	–	–	6	Lack of knowledge hinders willingness to use intervention	Prior knowledge about intervention improves uptake
Opportunity						
Social influences	–	–	–	10	–	Provider recommendation may impact patients' decision to use intervention
Intervention						
Cost	10	Potential cost and burden on patients deter providers from recommending intervention	–	10	Intervention cost deters patients from using intervention	Free trials provide flexibility
Evidence Strength & Quality	9	Lack of empirical evidence solidifies bias against intervention	Empirical evidence demystifies preconceived notions about intervention content	8	Lack of evidence discourages patients to try	Empirical evidence instills confidence
Adaptability	–	–	–	7	Lack of adaptability reduces user engagement	Intervention needs to be adaptive to patients' needs

n^a^ represents the number of respondents who reported on each construct

### Providers

As part of the Outer Setting domain of the CFIR framework, all 10 providers discussed at length patient stressors and needs as well as the lack of easily accessible mental health resources for cancer patients and survivors (*Patient Needs and Resources*). For example, Provider 1 said “We’ve seen an increase in depression and anxiety related to things going on in the world complicated by the survivorship issues, particularly if there are long-term late effects that people are also coping with”, adding, “We are in a mental health provider crisis, particularly for young adults, and particularly for young adults who do not have a lot of money to pay out of pocket… Once people are in survivorship, it becomes much more difficult to access those services” (see Table 2).

**Table 2:** Selected themes mapped on the CFIR + TDF frameworks (*N* = 20)

*Framework*	*Domain*	*Construct*	*Definition*	*Illustrative Quotes—Providers*	*Illustrative Quotes—Patients*
CFIR	Outer Setting	Patient Needs and Resources	Consideration of the needs and resources of the app's target group (e.g., perceived vulnerability, literacy, language, … availability of hardware, network connection)	“[There is] Anxiety around treatment, anxiety around … what the life looks like now. … missing out on school because you’re in treatment all the time. Not being able to see your friends; those are really big factors that impact mental health.” (Provider 1) "Finances certainly are big. Not having money and insurance. Some of our young adults don't have family or are not documented. So they have no insurance. They're working menial labor jobs." (Provider 8)	“I think … [my] PTSD is coming from the diagnosis, or an event similar to the cancer experience. … and certain thoughts and fears are…, oh my God, what if this lump is coming back … There’s always this thought like relapse and it’s a very common thought. It’s a very common fear.” (Patient 1)
CFIR	Inner Setting	Networks and Communication	High social capital within a team, i.e. dimensions of shared vision and information sharing, can contribute to effective implementation through a sense of "team spirit" or "community"	"You're in a team environment in our clinic; you have the patient care associates and the PCs and the regular nurses and nurse practitioners, and we all act together to get the patient through their visit." (Provider 4)	–
CFIR	Inner Setting	Culture	Norms and values, or a mindset and culture of an app provider	"We ultimately all have the same goal for these kids is to help them as much as we can during their treatment." (Provider 9)	–
TDF	Opportunity	Social Influences	Those interpersonal processes that can cause an individual to change their thoughts, feelings, or behaviors	–	“If my provider, my medical professional thinks that it’s best the interest for me to do it, I definitely would give it a try.” (Patient 9) “Coming from the hospital is like a plus. I think for me that would be enough.” (Patient 6)
CFIR	Intervention Characteristics	Cost	Costs of the app and costs related to the implementation of this app, including investment, supply and opportunity costs. Costs should be in relation to the expected benefits, both for the user and the app developer	"Money is often an issue and I don't want to burden the kids or the families." (Provider 6)	"I think cost is the biggest one [factor]." (Patient 7) "Just thinking about my medical bills before, even in the future, or if this were to happen again. Am I gonna be able to afford that?" (Patient 9)
CFIR	Intervention Characteristics	Evidence Strength and Quality	Description of the current status of previous, scientific findings on the quality and validity of the app	“There's the problem with the mobile apps as there's a million of them right. You have to have one that's been vetted.” (Provider 4) "Just be very concrete: This is what the scientific research is and the data that supports it, and that it truly can help, what parts do we not know for sure if it's helping or not, and just being very kind of scientific about it." (Provider 5)	If it really helps you, helps your mental state, then I’d be willing to pay however much because it's actually helpful.” (Patient 10) “I would say that I’d be more encouraged to use it if I could see some evidence behind it.” (Patient 2)
CFIR	Intervention Characteristics	Adaptability	Description of the extent to which core components of an app can be adapted, tailored, refined or newly developed to individual or local needs	–	"I do like whenever they explain the logic behind like why it works in a way we kind of understand, why are you doing what you're doing." (Patient 5) "The only thing I wish that some of the meditation sessions would be shorter towards the end. … I'm fidgety, my mind just wanders all the time. … I guess shorter bites of meditation will be better." (Patient 7)
CFIR	Characteristics of Individuals	Knowledge and Beliefs about the Intervention^a^	Perception of a credible external presentation of the app, as well as knowledge of the users how to handle it	–	"I think definitely mindfulness and meditation is helpful. It helps to track harmful thoughts and anxiety" (Patient 1) "If I was being diagnosed today, I think having a meditation app is probably good. … I think meditation should be a part of it [the treatment plan]." (Patient 4) "Mindfulness is a lot about kind of like reconnecting with your body and managing how you feel by being aware of like seeing in the moment and controlling how certain things affect you. 'Cause usually a lot of stuff is out of your control. So it's helping out with like realizing when you can and when you can't really control something. And then working to release whatever physical reactions you're getting." (Patient 5)
TDF	Motivation	Reinforcement	Increasing the probability of a response by arranging a dependent relationship, or contingency, between the response and a given stimulus	–	"Just having an article that lists Okay, you should do this, you should read for like five seconds, you should do this next, that's very boring. It's very monotonous, … but if there is a video or even a live person speaking…." (Patient 1)
CFIR	Process	Planning Engaging	Apps should be implemented according to plan & Attracting and involving appropriate people in the implementation and use of the app through a combined strategy of social marketing, education, role modelling, training and other similar activities	"I think there needs to be an introduction, or like a class or tutorials about what meditation is, what mindfulness is, what it's not. … We have regular meetings before we start our clinic, a huddle, where they go through what everyone is doing. So maybe if everyone else is talking about it [app], and you're the only one not doing it, you know, maybe you'll try it too." (Provider 4)	–

^a^Combined with *Knowledge* in TDF

*Cost*, part of CFIR’s Intervention domain, seems to overlap some of these concerns. For example, the cost associated with mental health care was another prominent topic mentioned by all ten providers. There was a shared understanding that for a mental health app to be useful to patients, it needs to be freely available to them (e.g., Providers 6 and 8). Another intervention-related factor discussed was *Evidence Strength and Quality*; providers discussed the need for evidence-based research on the validity of digital health apps. Some providers expressed skepticism regarding the evidence of the intervention content, in this case, mindfulness-based intervention: “Most people don't understand it, and physicians are going to be the exact same way. They're [physicians] going to have their preconceived biases and think it's about being like some hippie in the woods” (Provider 6). Furthermore, they note the importance of making evidence-based literature available to them: “There're articles in the medical journals that are about it… send in that kind of stuff to them [physicians] from time to time reminding them that we have the [mindfulness app] study that's available. Because often patients will take what the physician says as more relevant than what other people might say” (Provider 6 about physicians). Several providers stressed the importance of utilizing a “vetted” digital health intervention: “Meditation has a lot of solid research attached to it. Now it's more acceptable. They’re really gonna have to show it's evidence-based because they do not want to be associated with [lack of evidence], which is how most of what my mind body stuff is today” (Provider 1). In general, providers are receptive to learning about new evidence; Provider 10 discussed, “I would be happy to hear about the evidence behind a meditation app if there was ‘showcasing the evidence behind mindfulness is vital in its promotion…’ to really show that [it works is to say], look, this is the data, these are the trials, this is what people have studied, this is the outcomes. Because that at the end of the day is where we make most of our decisions. It's utilizing evidence-based medicine” (Provider 5).

As part of the Inner Setting domain (CFIR), providers strongly voiced the need for a supportive leadership and company culture (*Structural Characteristics* and *Culture*), open communication among the care team (*Networks and Communications*), and education about the implementation of such an app (*Readiness for Implementation*). For example, Provider 9 praised the formal and informal communication within the department (*Networks & Communication*): “We meet weekly to talk about our patients to see if anybody has any concerns and it’s a good way to sort of just make sure that we’re all on the same page.” Provider 8 also highlighted the importance of communication among care team members: “Because this is such a team approach that we do that even if it's a family that I resolve everything [with]… it's still something that I'm going to share with the rest of the team so that the next time they come in that they can be involved and be a support.”

*Available Resources* (Inner Setting) are another key element in the care and support of survivors: “We give that passport for care, with all their instruction sheets of all the potential late effects that can happen. There are things on mental health and post-traumatic stress there. I call it their ‘encyclopedia for health…’ And just leaving the door open, knowing that they can always call the social workers or the psychologist, and if they need assistance or things aren't working or going in the way they think and they need to come talk to somebody, the doors are open here” (Provider 3). Provider 4 shared “We have a lot of resources in this cancer institute. We have a licensed social worker for counseling onsite. We have a licensed psychologist. We have access to a young adult psychiatrist where we can prescribe medications” which further highlighted the strength of resources dedicated to the implementation and ongoing care of survivors.

Further, providers noted that the promotion of the app needs to be reinforced (*Reinforcement* in TDF) among the care team to help staff stay aware and informed of any changes and updates. Provider 1 pointed to the advantage of mental health apps as they are easily accessible, “even if they’re [patients] not with us,” however, cautioned that these services must be provided by “people who understand the impact of cancer on survivorship” which again spoke the direct relationship with the *Evidence Strength and Quality* of the app (Intervention Characteristic).

Given the multitude of digital health interventions broadly, and a large number of mindfulness specific apps, one cannot assume that healthcare providers would be familiar with all available supportive care tools and the evidence related to their efficacy. Education could potentially be a crucial element in successfully implementing the broad application of mindfulness apps within the hospital system. The entire care team including physicians, nurses, psychosocial providers, and other staff should be part of educational efforts to seamlessly introduce the app and to speak with ‘one voice’ when promoting the app to cancer patients (Provider 4). Provider 6 shared: “It is important for providers that not all of them [patients] necessarily are going to use it. They’re [patients] also going to have some misconceptions about meditation and mindfulness. Giving them some of the neuroscience behind it and the brain research that is done about it and giving them more of the hard facts as opposed to oh it's really helpful.” In sum, promoting and implementing an app hospital-wide would surely require education integrated with the Process domain as outlined in the CFIR framework. This highlights providers’ goal of “…teach[ing] our patients they have to advocate for their own health with their own healthcare providers. That's what we try and do… we're trying to do is to make them independent and taking care of their own health needs” (Provider 4).

### Patients

Like providers, all (*n* = 10) AYA survivors expressed the relevance of stressors and needs, sometimes years and decades after treatment (*Patient Needs and Resources*). Patient 8: “My biggest hurdle was… learning different coping mechanisms to deal with PTSD, experiencing flashbacks, dealing with hospital settings and things of that nature. I think all of that really affects you going forward and trying to get back to whatever your life will shape out to be when you’re done.” This is underscored by Patient 1 who shared that they are still “dealing with complications from late effects… even like 15 years after completed treatment because of my leukemia and side effects as a result.”

*Cost* is a consensus concern and survivors were cautious about their choices. Patient 7: “I think costs are the biggest one, time and for me personally depending on whether it's an app or [in]person location… I have little vision and I’m unable to drive, getting to some places is very either hard or expensive.” Free trials could be enticing and allow survivors to use the app without commitment; Patient 10 mentioned: “I feel like the best situation would be you kind of get a test trial. See what the app is like, and then go from there.” Aligned with Patient 9, survivors want to know if it does work before making any further commitment, which reflects the importance of *Evidence Strength and Quality*; Patient 1 suggested: “For me, it will be nice to see empirical evidence. Let's say this app out in the market is tailored just for survivors. If there is some research that let's say improves the mood of these patients or these survivors by, I don't know, about 12% or 20% versus prior to using the app, that might pique my interest a little bit more.” Patient 3 raised the rating aspect of apps: “I try to read all the comments to see how they will do. And then I would consider.”

Individual factors such as motivation are apparent. Patients emphasized the importance of *Reinforcement* (Motivation in TDF) in terms of app features and content. Patient 1: “It's about the app being interactive and engaging,” explaining further “having that interactive component I think is essential.” Another patient discussed the advantage of a “live” element: “I like a live chat… [where] we can really speak to another person and then meditate with them.” Another survivor: “I think it'll be really cool to have somebody else I can communicate with and meditate with” (Patient 3). Patient 4: “I think it would be helpful if there was a survivorship section not necessarily totally related to cancer. There's a lot of chronic diseases and illnesses that can go through with various degrees of daily impact on their daily lives. It doesn't necessarily have to be tailored to young adult cancer survivors. In my opinion, maybe people who have chronic medical issues or diagnoses who are young adults… It would be good to have people who have that history. As a survivor, or as a person who has a complex medical background, I think separating it by different stages of life as well would be helpful.”

Additionally, the trust survivors have in their clinicians makes them more amiable in giving mindfulness a chance (*Social Influence* in TDF). For example, Patient 4 noted, “I would say if my doctor and oncologist’s office suggested it and recommended it, that’s good enough for me.” Patient 8 shared: “[If the] Doctor tells me, ‘Hey, I think this would be helpful,’ I'm gonna try it.” Their trust in their clinical providers (*Social Influence*) offset concerns over other important factors (i.e., *Evidence Strength and Quality* shared by both providers and patients).

In discussing Intervention Characteristics (CFIR domain), Patient 10 expressed the advantages of digital health apps (versus going to an in-person appointment; *Relative Advantage*): “There’s a lot of things that you need to remember [as a cancer survivor]. It’s almost like this constant additional stressor, in addition to all of the other things that you have to do in terms of being alive. That sometimes I do feel like it’s like the straw that broke the camel’s back. So having that [an app] as an alternative avenue to kind of make sure that you’re taken care of and accounted for up to that point would probably be helpful.” AYA survivors also highlighted the need for the app to be adaptable (*Adaptability*), such as having reminder features (Patient 8), adjustable timeframes (Patient 7), progress tracking, and explanations that provide the logic behind mindfulness (Patient 5). Others expressed that the app should allow for opportunities to connect with others (i.e., forum options; Patient 6). Patient 3 voiced “I think it’d be really cool to have somebody else I can communicate with and meditate with them… [where] I just feel like I’m in a community with everyone who is in the same situation.” These responses elucidate the unique experiences (Patient 5) of cancer survivors. In the context of *Social Influence*, the ability to connect with others could improve the use of meditation apps. Patient 4 raised the opportunity for digital health interventions to also serve as a support for the broader family system: “It's a stressful time and not just for the patient themselves, but for any family member that's directly involved or a spouse. It's more than just the individual going to treatment. I think the people who are in that immediate bubble, like immediate family and members, would also benefit from that as well.”

## Discussion

The goal of this study was to identify key patient-, provider- and organization-level barriers and facilitators to implementing supportive care through digital health interventions in AYA survivorship care. The findings suggest that some factors related to acceptability and adoption were both shared by providers and survivors, while others were participant-specific. Both providers and patients described *Patient Needs and Resources* (Outer Setting)*, Cost* (Intervention Characteristics), and *Evidence Strength and Quality* (Intervention Characteristics) as priorities when it comes to factors influencing their adoption and use of mobile app interventions. These factors were also found in prior studies; for example, a recent scoping review found that 10 articles suggested *Cost* is a barrier to the adoption of digital health interventions [42]. In addition to the overall cost of the app [43], other expenses such as having a device [44] and paying for internet data [45, 46] could be potential barriers for patients when deciding to use digital health interventions. Although not explicitly mentioned, clinical providers have concerns over the time and effort it takes to integrate digital health interventions into practice as well as workflow [47] because these investments are often not reimbursed [48–51] which potentially speaks to the larger *External Policy and Incentives* (Outer Setting). For example, medical personnel (i.e., physicians and nurses) will not be reimbursed when providing psychosocial services. Thus, it is important to collaborate with the mental health team (i.e., social workers and psychologists) to support supportive care. This reflected the importance of the interdisciplinary approach in the context of the Inner Setting (CFIR): *Networks and Communications*, *Structural Characteristics,* and *Culture* as suggested by many providers.

The content and evidence of efficacy for psychological interventions are not always apparent to providers. For mindfulness, skepticism is well documented [52] and could be a barrier to influencing its use and adoption by both providers and patients (*Evidence Strength and Quality*). Our results showed that providers believed education is a crucial element in successfully implementing supportive care, especially for meditation mobile apps, given the skepticism toward mindfulness as an intervention. The interdisciplinary team including physicians, nurses, psychologists, social workers, and staff should be a part of the educational efforts to seamlessly introduce mindfulness and other digital health interventions. Prior research has suggested implementing mindfulness training sessions for new employees or as a continuing education option offered to staff [53]. Education can demystify any uncertainty about the interventions and provide evidence and support, thus enhancing buy-in. Buy-in from providers can also instill patient confidence in the intervention. This coincides with *Social Influence* (TDF), where our AYA survivors expressed their trust in their providers when recommending any interventions. Provider recommendation has been found to be a strong predictor of uptakes in other interventions and preventions [54, 55]. This aligns with prior studies about the importance of the patient-physician relationship [42]. In addition, the trust patients have in their providers can perhaps overcome doubts or skepticism that patients may have about the evidence in support of the intervention (*Evidence Strength and Quality*). Thus, educating providers and ensuring their buy-in may have additional benefits. Similarly, educating patients about the evidence in support of mindfulness or other interventions can answer any misconceptions about the practice (*Knowledge*). *Knowledge* is an important factor as suggested by previous studies; for example, wrong perception [44–46, 56] and a lack of knowledge [44, 57–60] were seen as problematic to mobile health usage. However, time and effort could present as barriers due to the lack of reimbursement structure for extended patient education about supportive care by medical providers (*Cost* and *External Policy and Incentives;* [48–51]). However, it is important to note that this discussion regarding *Cost and External Policy and Incentives* only reflects the unique hybrid multiple-payer healthcare system in the United States. Other systems (e.g., Single-Payer and State-Based Financing) may host different structural and financial barriers to the implementation of digital innovations.

Although there were common factors shared by clinical providers and survivors, there were noticeable differences among them. In general, providers discussed structural/contextual (i.e., *Inner* and *Outer Settings*) and intervention-related (*Intervention Characteristics*) factors impacting adoption and workflow which in turn influences patient care. For example, providers highlighted the need to use an interdisciplinary team approach to handling patients’ psychosocial needs reflecting the importance of *Culture* and *Networks and Communications* (Inner and Outer Settings). Survivors, on the other hand, focused on intervention- and individual-related factors. In addition to aspects of the intervention adaptability and evidence of effectiveness, survivors also spoke about how *Reinforcement* and *Knowledge* may assist their adoption and sustained usage of digital innovations. It is no surprise that the groups differ in their perspective on the importance of factors related to the acceptability and adoption of digital health apps, supporting our rationale to include multiple key stakeholders in this work. Specifically, survivors were likely to focus on the individual as well as intervention factors because those are the most relevant concerns directed at their own behaviors. On the other hand, the providers’ frame of reference is based on their views on how well digital health interventions can be integrated into their workflow and meet the needs of their patients. Workflow integration demands contextual and structural compatibility (e.g., supporting clinic infrastructure [61]) which is manifested in *Inner* and *Outer Settings*.

In this study, we focused on identifying determinants of the acceptability and adoption of digital health interventions among providers and AYA in survivorship care in the United States. In addition to acceptability and adoption, there are other important implementation outcomes (e.g., appropriateness, feasibility, fidelity, implementation cost, penetration, and sustainability) [62] that should be considered in future work. Implementation processes and outcomes are dynamic, complex, and interrelated [63–66], and they often change throughout the implementation process [62]. The determinants can change based on the maturation of the implementation process (e.g., sustainability vs. early adoption).

### Study limitations

Strengths of this work include using a multiple stakeholder perspective to understand factors relevant to implementing digital health interventions in AYA survivorship care and the use of a theory-driven framework [31] in design and analysis. There are also several limitations to our study. Our sample was a convenience sample with a relatively small sample size. We had a predominantly white, educated, female sample. This aligns with the literature that racial/ethnic and low SES participants [67, 68] are often underrepresented in research. Also, the AYA survivors in the study were emerging adults aged between 18 and 29 years, and therefore, our conclusion may not be relevant to older adolescents (age 15–17) who may not yet be fully responsible for their own care or independently paying for supportive care interventions such as mobile apps. All the AYA participants were also referred from a previous meditation mobile app study. Their existing experience with meditation mobile apps may not reflect the general population. However, we believe this experience could give participants better insights into how it may be incorporated into their day-to-day lives. Similarly, all the providers and staff were recruited from Rutgers which may only reflect other similar healthcare systems. Future studies should focus on enrolling participants from historically underrepresented backgrounds; strategies include: 1) Developing participant-centered recruitment strategies to identify and address barriers to participation (e.g., addressing patients’ concerns); 2) forming relationships with providers who can potentially help with enrollment; and 3) building trust and foundations for community involvement [69]. Other tangible strategies include hiring diverse staff to assist with recruitment [69] and addressing individual barriers such as transportation [70]. Lastly, although we employed a multiple-stakeholder approach, we did not include possible external influences such as health insurance companies and policymakers who have a better understanding of the Outer Setting. Future work should explore the policy-level factors with the intention of developing implementation strategies targeting factors such as *Cost*.

### Clinical implications

Although there are limitations to the findings, we can harness results from this group to inform how we should approach addressing factors related to implementing digital health interventions. For example, the results showed that the empirical evidence in support of the intervention was important to our college-educated AYA sample. When developing or selecting mobile interventions for implementation, researchers should consider paying attention to specific characteristics of the targeted populations, such as those with higher education, and apply tailored strategies to address their concerns and needs, such as referencing empirical evidence in participant recruitment materials.

The results from this study can inform the development of implementation strategies to improve the adoption of mindfulness mobile apps and other digital health innovations in AYA survivorship care. For instance, implementation strategies may focus on education to target *Knowledge* and *Evidence Strength and Quality*. For example, we can utilize interactive group training [71], performance-related feedback [71], and opinion leaders [71] to improve knowledge, skills, and attitudes in training sessions among staff. Post-training, we can provide champions/clinical supervision [71] and make guidelines and resources pertaining to supportive care available to providers (i.e., dissemination of training materials [71]). Similarly, providers can serve as champions [71] to recommend and promote patients’ usage. In a structural approach, evidence-based apps can be incorporated into Electronic Medical Record (EMR) workflows; for example, EMR could include built-in process reminders to encourage digital health intervention adoption [71] when providers conduct mental health screenings with their patients. Digital health interventions [51] can be enhanced by working with developers to tailor mobile apps specifically to AYAs (e.g., provide cancer-tailored content, offer free trials, etc.).

## Conclusions

Using mindfulness-based mobile apps as a case example, this qualitative study explored the potential barriers and facilitators of implementing digital health interventions in AYA survivorship care. Potential facilitators and barriers to digital health intervention adoption were identified that could help guide the development of implementation strategies to promote and sustain the adoption of these digital interventions. Overall, the findings suggest that while AYA survivors reported that both individual and intervention characteristics influenced their adoption or use, clinical providers were more concerned about intervention and contextual factors related to the implementation of digital health interventions in survivorship care. Future research should continue to explore policy-level factors such as insurance payors and policymakers while aiming to develop and test implementation strategies related to individual and contextual factors.

### Supplementary Information

Supplementary Material 1.

Supplementary Material 2.

## Data Availability

The data that support the findings of this study are available from the corresponding author upon reasonable request. The data are not publicly available due to privacy or ethical restrictions.

## Abbreviations

AYA: Adolescent and Young Adult
CFIR: The Consolidated Framework for Implementation Research
TDF: Theoretical Domains Framework
