# Supplementary material for: Understanding the multilevel factors influencing the implementation of digital health interventions for supportive care in Adolescents and Young Adult (AYA) cancer survivorship: determinants of adopting mindfulness-based mobile applications
Source: Implement Sci Commun. 2024 Jul 17;5:76. doi: 10.1186/s43058-024-00612-w (PMC11253365; doi:10.1186/s43058-024-00612-w)
Supplement: Supplementary file 2 — Supplementary Material 2. [file 43058_2024_612_MOESM2_ESM.docx]

**Interview Guide**

**Title: Understanding the Multilevel Factors Influencing the Implementation of Mindfulness-based Mobile Applications in Adolescents and Young Adult (AYA) Cancer Survivorship Care**

***NOTES TO INTERVIEWER:***

The goal of this interview guide is to *understand how AYA cancer survivors and their providers make decisions on adopting/using mindfulness-based meditation apps and to a larger extent, mHealth psychosocial interventions.*

**Introduction:**

Thank you for taking the time to speak with me today. We are interested in your ideas about mental health care for young adult cancer survivors and the potential role of mobile apps such as meditation apps for improving mental health. In particular, we are interested in how mobile app interventions can be integrated into survivorship care in clinical settings. With the information from this study, we hope to develop more effective strategies to increase the use of meditation mobile app. Our discussion should last between 30 to 45 minutes. All of your responses will remain confidential. You may choose to stop the interview at any time, and there is no penalty to you for not completing the interview.

(If the participant is a university employee, add: Taking part in this research is not a part of your university duties, and refusing will not affect your job. You will not be offered or receive any special job-related consideration if you take part in this research.)

Before we begin, we would like to ask your permission to audio record our discussion. The interview will be transcribed, however, your name or any personal identifiers will not be associated with any of the notes. The audio recordings will be deleted once the project is complete. Would it be OK with you if I record this interview? [Yes – proceed with recording] [No – do not record]

Do you have any questions before we begin? I will be asking you a number of questions regarding mental health care with AYA cancer survivors in their survivorship care. Mental health care refers to any formal or informal service to improve emotional health and well-being, such as individual or group therapy, referral to social workers/psychiatrists/psychologists, spiritual counseling, or recommending resources (e.g., books, mobile apps, etc.),

GENERAL PSYCHOSOCIAL/MENTAL HEALTH PRACTICE:

Providers + Administrators

- What do you think are the major psychosocial/mental health risks for AYA cancer survivors?
- How important is mental health care in AYA survivorship care?
- What are oncology providers’ roles (e.g., oncologists, nurses, etc.) in the mental health care of AYA cancer survivors?
- When should mental health care take place? (Prompt: How do you screen AYA cancer survivors for mental health?)
- *What influences you to provide or refer survivors for mental health care? (*Remember to check CFIR: Individual characteristics/TDF domains*)
- *Are there structural/organizational or outside factors that influence your decision to provide mental health care? (*Remember to check CFIR: Outer and Inner Setting*)

Patients + Family Members

- What do you think are the major psychosocial/mental health risks for young cancer survivors?
- What do you think is the role of your medical team (i.e., oncologists, nurses) in addressing mental health needs? Would you prefer to receive mental health services as part of survivorship care or separate from it?
- When should mental health needs be addressed? By whom? How often? (e.g., at every survivorship visit, at certain timepoints only?)
- *What would influence you to follow-up with a mental health referral or service offering from your medical team? (*Remember to check CFIR: Individual characteristics/TDF domains*)
- *What barriers are there for getting mental health care services? (*Remember to check CFIR: Outer and Inner Setting*)

THOUGHTS ON MEDITATION AND MOBILE APP

Now I’d like to ask you some questions about mindfulness-based meditation interventions.

Providers + Administrators

- What do you know about mindfulness-based meditation or other Complementary and Alternative Medicine (CAM)^[[1]](#footnote-1)^ approaches? How useful do you think they are for patients? How have or would these approaches fit into your work?
- In recent years, many behavioral health interventions have moved to delivery via the internet or mobile apps. What are your thoughts about mobile app interventions for mental health? How relevant are they for AYA cancer survivors? How would a mindfulness-based meditation mobile app fit into AYA survivorship care?
- *What factors would influence you to recommend a meditation mobile app or other mHealth app for AYA survivors? (*Remember to check CFIR: Intervention Characteristics*)

Patients + Family Members

- What do you know about mindfulness-based meditation or other Complementary and Alternative Medicine (CAM)1 approaches? Have you ever tried any? [If so, tell me about your experience] How would these approaches fit into survivorship care?
- There are a number of mobile apps that offer mental health services, such as telehealth therapy or mindfulness-based meditation (e.g., Calm, TenPercentHappier, Headspace). What are your thoughts on mobile app interventions for mental health? How would a mindfulness-based meditation mobile app fit your survivorship care?
- *What factors would influence you to use a meditation mobile app or other mHealth app for mental health need? (*Remember to check CFIR: Intervention Characteristics*)

****NOTE TO INTERVIEWER: Below, is a checklist of TDF domains and CFIR constructs, with example prompts for each. As participant responds to above questions, using the blank column to the right to check off items that they address. After the participant responds, prompt them to address any checklist item which they may not have already addressed. You don’t need to ask all domains and constructs, but be mindful which domains have not been addressed and see if the checklist questions are relevant to participants’ responses.***

| **CFIR: INDIVIDUAL CHARACTERISTICS /**  **TDF DOMAINS** | | |  |  |  |
| --- | --- | --- | --- | --- | --- |
| **Questions to Providers/Admins** | | |  | **Questions to Patients/Families** |  |
|  | **Knowledge (TDF)/ Knowledge and beliefs about the intervention (CFIR)** | Are there clinical guidelines that make recommendation regarding mental health interventions^[[2]](#footnote-2)^ for AYA cancer survivors?  What is the role, if any, of clinical guidelines for making recommendations about mental health intervention for AYA cancer survivors?  Prompts: How do you see digital interventions fitting into clinical guidelines? |  | Are you aware of any help including programs or hospital staff that are offered to you to help with your stress?  What do you know about using mobile app to help managing with stress? |  |
|  | **Skill (TDF)** | What processes are in place to identity AYA survivors with distress?  What do you normally do when AYA cancer survivors who presented with distress?  Prompts: What skills are required or have you obtained in your training to handle patients in distress?  What do you do when AYA cancer survivors and their families ask you for psychosocial help? |  | What do you normally do if you are overwhelmed by stress?  Are there any skills you have learned to handle stress? |  |
|  | **Beliefs about capabilities (TDF)/ Self-efficacy (CFIR)** | How confident are you in recommending mental health interventions to patients, especially AYA survivors?  Prompts: problems you may encounter/additional expertise or experience needed |  | How confident are you in managing your own stress/mental health? |  |
|  | **Beliefs about consequences (TDF)** | What are the potential benefits or disadvantages to provide mental health interventions to AYA cancer survivors?  What do you think the consequence (if patient's stress/mental health is not managed) would be in the context of your work? |  | What are the potential benefits or disadvantages if your stress is not managed?  If not managed, how is it going to impact your survivorship care? |  |
|  | **Motivation & goals (TDF)** | How important is it to help manage AYA cancer survivors' mental health? What influences the likelihood of providing mental health intervention? |  | How important is it to manage your stress?  How important is it that their survivorship care team help manage their stress? |  |
|  | **Memory, attention, and decision processes (TDF)** | When AYA survivors present with mental health symptoms, what, if any, are the tasks that you do automatically?  What factors do you consider when making the decision about offering mental health care to your AYA survivor patients?  Prompts: What are the factors related to your patients specifically that may influence your decision? What about factors outside your practice?    Prompt: time, workload, patient history, policy… |  | Say if the hospital or provider is recommending you a mobile app or referring to you a professional for help in managing stress, what will you do and why?  What factors are important to your decision making? |  |
|  | **Environmental context and resources (TDF)** | What physical or environmental resources would influence how you provide mental health interventions?  Prompt: reimbursement? standing orders? availabilities of other resources? patient’s insurance coverage? |  | What physical or environmental resources would influence you getting help with managing your stress? |  |
|  | **Social Influences (TDF)/ Individual Identification with the organization (CFIR)** | How might views or opinions of others, such as colleagues, patients, professional groups, or others in your practice influence whether or not you offer mental health interventions for AYA survivors?  Is there consensus in the profession about your role in patients' mental wellbeing? |  | How might views or opinions of others, such as peers, colleagues, and family members influence whether or not you receive help for your managing your stress?  What is the general consensus of the people around you regarding getting help for managing stress?  What is their opinion about using mobile apps for managing stress/mental health? |  |
|  | **Emotion (TDF)** | How might your emotions influence whether or not you offer mental health interventions to your AYA survivors? |  |  |  |
|  | **Behavioral regulation (TDF)** | Do you track patients’ distress over time? (How do you follow up with patients if you refer them for mental health care?) |  |  |  |
| **CFIR: OUTER SETTING** | | |  |  |  |
| **Questions to Providers/Admins** | | |  | **Questions to Patients/Families** |  |
|  | **Patient needs and resources** | What mental health interventions do AYA survivors need? What barriers do AYA survivors face in accessing them? |  | To what extent do your providers address your mental health/wellbeing? |  |
|  | **Cosmopolitanism** | Do you have a network of mental health providers you would refer to?  How do you learn about mental health intervention information for AYA survivors? Prompts: Professional networks? Local or national conferences? Social media? |  |  |  |
|  | **Peer Pressure** | To what extent are you aware of other hospitals providing mental health care as part of survivorship care?  Do you think your colleagues in other practices/hospitals provide mental health interventions to their AYA survivors? |  |  |  |
|  | **External policy & incentives** | Are there any local or national guidelines that play a role in whether or not you provide mental health interventions to your AYA survivors?  Are there financial incentives provided by your practice or another organization that would influence whether or not you provide mental health interventions to your AYA survivors? |  |  |  |
| **CFIR: PROCESS** | | |  |  |  |
| **Questions to Providers/Admins** | | |  | **Questions to Patients/Families** |  |
|  | **Engaging** | Are there key influential individuals that would affect whether you or clinicians in the hospital decide whether or not to provide mental health interventions to your AYA survivors?  Prompts: Is there a champion who are promoting mental health service provision? |  |  |  |
| **CFIR: INNER SETTING** | | |  |  |  |
| **Questions to Providers/Admins** | | |  | **Questions to Patients/Families** |  |
|  | **Structural characteristics** | How do you think the size and maturity of your program affects how clinicians decide whether or not to provide mental health interventions to your AYA survivors? |  |  |  |
|  | **Networks & communication** | How do you typically find out about new information within the hospital related to mental health services? How would information about guidelines regarding mental health interventions for AYA survivors be shared in here? |  |  |  |
|  | **Culture** | Are there any aspects of the hospital’s culture (general beliefs, values, assumptions that people embrace) that affects whether or not you decide to provide mental health interventions for AYA survivors? |  |  |  |
|  | **Implementation climate** | Do you think that there is a strong need to change mental health practices for AYA survivors here?  Are there standard work processes and practices regarding providing mental health interventions for AYA survivors in the hospital?  Does providing mental health interventions for AYA survivors conflict with other priorities in the hospital? |  |  |  |
|  | **Readiness for Implementation** | How are the leaders’ commitment to making changes to mental health practices for AYA survivors here?  What are the resources available to making changes to the mental health practices for AYA survivors here? Are there accessible information for the staff here? |  |  |  |
| **CFIR: INTERVENTION CHARACTERISTICS** | | |  |  |  |
| **Questions to Providers/Admins** | | |  | **Questions to Patients/Families** |  |
|  | **Innovation source** | A lot of meditation mobile apps are commercially available. Would you prefer recommending these commercially available apps to AYA patients or would you prefer something developed by our researchers? |  | A lot of meditation mobile apps are commercially available. Would you prefer using these commercially available apps or would you prefer something developed by the researchers from the hospital? |  |
|  | **Evidence strength and quality** | What are your opinions about meditation mobile apps? What do you think the patients think of it?  What evidence are you aware of that shows whether meditation mobile apps will work for our AYA survivor population?  What kind of supporting evidence or proof is needed about the effectiveness of meditation mobile app to get other staff on board? |  | What are your opinions about meditation mobile apps? What do you think your providers or hospital staff think of it?  What evidence are you aware of that shows whether meditation mobile apps work?  What kind of supporting evidence or proof is needed to get you on board? |  |
|  | **Adaptability** | Are there any changes that need to be made to meditation mobile apps so they will work effectively for AYA survivors? |  |  |  |
|  | **Complexity** | How complicated do you think meditation mobile apps are?  How complicated is it to implement meditation mobile apps into AYA survivorship care? What do you think the steps are? |  | How complicated do you think meditation mobile apps are? |  |
|  | **Relative Advantage/Cost** | How do meditation mobile apps compare to other similar existing programs or interventions in this hospital or elsewhere?  Is there another intervention that the hospital staff would rather implement?  Any there cost concerns for you or the patients? What is an appropriate price for patients?  What factors about meditation mobile apps will impact the adoption (using it with patients) and maintenance (continue using it)? |  | How does a meditation mobile app compare to other interventions you know?  Is there another intervention that you would rather try?  Any there cost concerns for you? What is an appropriate price for you?  What factors about a meditation mobile app would make you want to try it? What would make you want to keep using it? |  |
|  | **Design Quality & Packaging** | What feature(s) must a meditation mobile app have? Anything on the interface? Usability? |  | What feature(s) must a meditation mobile app have? Anything on the interface? Usability? |  |
|  | **Trialability** | How important would piloting with a small group of patients first before making it available to everyone? |  |  |  |
| **Potential questions** | | |  |  |  |
| **Questions to Providers/Admins** | | |  | **Questions to Patients/Families** |  |
|  |  | Are there procedures or ways of working that would make it easier to offer mental health intervention?  What do you think would have to change at your practice to determine the need to provide mental health interventions for AYA survivors?  Can you identify any training needs or guidelines regarding mental health that need to be more specific?  (Prompts: EMR alerts for best practices, pop-up notifications)  Do you think AYA survivors prefer receiving mental health interventions from their oncology teams or outside of survivorship care? |  |  |  |

Those are all of the questions that I had planned for today. Is there anything else that you feel is important that we haven’t yet covered?

1. Mindfulness-based meditation or other Complementary and Alternative Medicine (CAM) were referred to as evidence-based interventions. [↑](#footnote-ref-1)
2. Including direct mental health intervention, referral to social workers/psychiatrists/psychologists, recommending resources (e.g., books, mobile app, etc.) [↑](#footnote-ref-2)
